# A Protocol of National Mixed‐Methods Assessment of Childhood and Maternal Immunization

**DOI:** 10.1002/hsr2.72566

**Published:** 2026-06-07

**Authors:** Seyed Aria Nejadghaderi, Paria Dehesh, Mehran Nakhaeizadeh, Mohammadreza Rajabalipour, Faeze Tahrudi, Naser Nasiri, Seyed Mohsen Zahraei, Hamid Sharifi, AliAkbar Haghdoost

**Affiliations:** ^1^ HIV/STI Surveillance Research Center, and WHO Collaborating Center for HIV Surveillance, Institute for Futures Studies in Health Kerman University of Medical Sciences Kerman Iran; ^2^ Knowledge Hub for Migrant and Refugee Health, Institute for Futures Studies in Health Kerman University of Medical Sciences Kerman Iran; ^3^ Social Determinants of Health Research Center, Institute for Futures Studies in Health Kerman University of Medical Sciences Kerman Iran; ^4^ Department of Biostatistics and Epidemiology, Faculty of Public Health Kerman University of Medical Sciences Kerman Iran; ^5^ Modeling in Health Research Center, Institute for Futures Studies in Health Kerman University of Medical Sciences Kerman Iran; ^6^ Bio Environmental Health Hazards Research Center Jiroft University of Medical Sciences Jiroft Iran; ^7^ Center for Communicable Disease Control, Ministry of Health and Medical Education Tehran Iran; ^8^ Institute for Global Health Sciences University of California San Francisco California USA

**Keywords:** immunization coverage, Iran, mixed‐methods, vaccination survey, vaccine equity

## Abstract

**Background and Aims:**

Accurate measurement of routine immunization coverage is essential for detecting immunity gaps, guiding programmatic responses, and supporting equitable vaccine delivery. Iran's Expanded Programme on Immunization has achieved high coverage, but administrative estimates can mask some disparities. The aim of this protocol is to evaluate coverage among children under 3 years old and mothers who have given birth within the last 12 months.

**Methods:**

We will conduct a national, cross‐sectional, mixed‐methods survey on vaccination coverage across all 31 provinces of Iran. A multistage, stratified cluster design will target about 9200 children aged 0–35 months (1325 clusters) and 2109 mothers who delivered in the past 12 months (211 clusters), with oversampling of smaller strata and an allocated proportion for non‐Iranian residents. Vaccination status will be verified preferentially from home‐based records and electronic registries, supplemented by caregiver recall. Digital data capture and photographed records will be used alongside supervisory quality checks. Quantitative analyses will apply complex survey weighting to estimate coverage, dropout, timeliness, and missed opportunities. Also, thematic analysis of 30–40 in‐depth interviews will show determinants of vaccination.

**Results:**

This manuscript describes the protocol for a national mixed‐methods study. No empirical results are presented.

**Conclusions:**

The findings from this mixed‐methods study will deliver high‐quality, disaggregated evidence on immunization coverage and its drivers in Iran, enabling policymakers and programme managers to design and implement targeted interventions to close equity gaps and support sustained uptake of both established and newly introduced vaccines.

## 1 Introduction

The Expanded Programme on Immunization (EPI), launched by the World Health Organization (WHO) in 1974, established the global foundation for delivering essential vaccines to children and remains central to efforts aimed at reducing vaccine‐preventable morbidity and mortality worldwide [1]. Over subsequent decades, the coverage of major childhood vaccines, including Bacillus Calmette‐Guerin (BCG), diphtheria–tetanus–pertussis (DTP), polio, and measles, has increased in many regions, with routine indicators such as the third dose of DTP (DTP3) becoming standard performance metrics for national programmes [2]. Immunization coverage is necessary as the proportion of the target age group who have received a given vaccine dose, and many countries calculate administrative coverage by dividing doses reportedly administered by estimated population denominators. However, these administrative estimates are vulnerable to numerator and denominator errors and therefore require external validation [3]. Because of these known limitations, the WHO and immunization partners emphasize the complementary role of household coverage surveys, which verify vaccination status through home‐based records, caregiver recall, and facility records, to provide more accurate and actionable coverage estimates [2, 4].

The national EPI in Iran, which was formally introduced in the 1980s, has been integrated into the country's primary healthcare system and has reported high coverage levels that contributed to major public health achievements, including interruption of poliovirus transmission and substantial reductions in measles and neonatal tetanus incidence [1, 5]. The national routine schedule, which provides vaccines free of charge, includes BCG, a birth dose of hepatitis B and bivalent oral polio vaccine (bOPV) at birth, pentavalent and bOPV series beginning at 2 months with inactivated polio vaccine (IPV) introduced alongside later doses; and measles, mumps and rubella (MMR) doses in the second year of life, among other recommended vaccines incorporated into the EPI platform [1, 6]. National and subnational monitoring have shown high aggregate coverage, but administrative figures alone can mask heterogeneity across provinces and population subgroups, necessitating periodic, rigorous assessment to detect pockets of under‐immunization [5, 6]. Even internally validated administrative systems require complementary evaluation to ensure that programmatic decisions and resource allocation are guided by measurements that accurately reflect population‐level immunity [3, 6].

Despite strong systems and a data quality self‐assessment that found acceptable validity in routine administrative records, immunization data remain susceptible to a range of errors at the point of recording, during aggregation, and in population estimates, which can produce both over‐ and under‐estimates of true coverage [6, 7]. International partners have therefore developed systematic tools to assess and improve data quality, including the Data Quality Audit and the Data Quality Self‐Assessment, and the WHO has published detailed guidance for vaccination coverage surveys to standardize design, sampling, and analysis across settings [8, 9, 10]. Coverage surveys also provide programmatic information, such as dropout rates, the prevalence of zero‐dose children, timeliness of vaccination, and missed opportunities for simultaneous vaccination, which cannot be reliably inferred from administrative data alone [10, 11, 12].

Demographic and socioeconomic dynamics, including internal inequalities and inflows of migrants and refugees from neighboring countries with less robust health systems, have highlighted the need for up‐to‐date, disaggregated coverage estimates. These equity gaps, particularly among rural populations and non‐Iranian residents, are often driven by behavioral and social drivers of vaccine uptake, such as limited physical and financial access in remote areas, language and cultural barriers, vaccine hesitancy linked to misinformation or mistrust in health services, and differing community norms around preventive care [5, 13]. Recent national survey data indicated overall high coverage among Iranian children but lower coverage among non‐Iranian residents, a disparity that risks localized immunity gaps and outbreak potential if unaddressed [5]. To inform policy, strengthen program responsiveness, and support introduction and sustained uptake of newer vaccines such as pneumococcal conjugate and rotavirus, a mixed‐methods evaluation that combines WHO‐aligned coverage survey methods with Behavioral and Social Drivers (BeSD) tools is warranted. Such an approach improves both measurement accuracy and understanding of the demand‐ and supply‐side determinants of immunization [10, 14]. The present protocol therefore aims to specifically: (1) estimate the coverage rates for routine vaccines at the national and provincial levels, disaggregated by sex, residence (urban/rural), and nationality (Iranian vs. non‐Iranian) in Iran; (2) quantify discrepancies between administrative coverage data and survey‐based estimates; (3) identify behavioral and social drivers associated with incomplete or delayed vaccination among children and mothers; and (4) explore contextual barriers and facilitators influencing vaccine uptake through in‐depth qualitative interviews with caregivers, health workers, and community leaders.

## 2 Methods

### 2.1 Study Design and Setting

This study is a national, cross‐sectional, mixed‐methods vaccination coverage survey designed to estimate routine immunization coverage among children under 3 years of age and maternal vaccination coverage among women who gave birth in the previous 12 months across all 31 provinces of Iran. The survey follows WHO vaccination coverage survey guidance for design, sampling, data collection, and analysis, and integrates the WHO BeSD tools to capture demand‐ and supply‐side determinants of vaccine uptake [10, 14].

### 2.2 Theoretical Framework

The behavioral component of the study is guided by established models of health behavior, including the COM‐B (Capability, Opportunity, Motivation–Behavior) model and the Health Belief Model (HBM). These frameworks provide a structured lens to interpret the BeSD domains' confidence, social processes, practical access, and motivation. According to the COM‐B model, vaccination uptake results from the interaction between an individual's capability to act, the opportunities provided by the system and environment, and the motivation to perform the behavior. Integrating these models ensures that qualitative and quantitative analyses move beyond descriptive statistics to identify actionable behavioral determinants for intervention design. A large‐scale survey alone, while excellent for measuring coverage levels and statistical associations, cannot capture the nuanced contextual explanations, lived experiences, or complex social and structural drivers that shape vaccine decisions, particularly among diverse and underserved populations. The qualitative component is therefore essential to answer the critical “why” questions that quantitative data alone leave unanswered.

### 2.3 Study Population and Sampling

The primary quantitative population comprises children aged 0–35 months residing in selected households and mothers who delivered a live birth in the preceding 12 months, with eligibility defined according to WHO cluster survey age windows and national registry conventions [5, 10]. The qualitative component will purposively sample parents, health‐care providers, community leaders, non‐Iranian residents, and policymakers to explore barriers and enablers to vaccination uptake, with an intended sample of approximately 30–40 in‐depth interviews to reach thematic saturation [5, 14].

A multistage, stratified cluster sampling design will be used with provinces allocated into four strata (population < 1.5 million; 1.5–5 million; > 5 million; and Tehran as the capital with > 14 million population) to ensure adequate representation from less‐populated and potentially underserved areas and to enable precise provincial and national estimates. To minimize potential subtle biases in the representation of specific subpopulations (particularly non‐Iranian residents), the disproportionate allocation explicitly oversamples smaller strata and includes a pre‐specified proportion of clusters with higher expected non‐Iranian density. Sensitivity analyses will later assess the impact of this design choice. Sampling allocation across the four provincial strata is summarized in Table 1. The table presents the distribution of the total 9200 target interviews across 31 provinces grouped into four population strata, including the number of clusters per province, average households per cluster, and design parameters used for sample size estimation. The details of the sample size calculation are provided in Supporting Information S1: Appendix 1.

**Table 1:** Sampling design and allocation across population strata in the national vaccination coverage survey, Iran.

Population stratum	Provinces included	Completed child interviews	Estimated households to visit	Clusters per province (avg)	Total clusters	Avg. households per cluster^a^
< 1.5 million	13	2983	4972	2–3	429	117
1.5–5 million	13	3961	6597	3–4	572	117
> 5 million	4	1727	2877	15–16	248	117
Tehran (> 14 M)	1	530	882	76	76	117
Total	31	9201	15,329	—	1325	≈117

*Note:* Sampling followed a disproportionate stratified cluster design to ensure adequate representation of small and deprived provinces. Estimates are based on the WHO 2018 Vaccination Coverage Survey Manual parameters (DEFF = 3.27, ICC = 0.33, m = 7, non‐response = 10%).

^a^ Average number of listed households required to locate approximately 7 eligible children per cluster.

The assumed parameters for the intracluster correlation coefficient (ICC = 0.33), design effect (DEFF ≈ 3.27 for the child survey and 5.96 for the maternal survey), coefficient of variation (CVw = 0.3–0.7), and non‐response adjustment factor (~1.11) were derived from the WHO Vaccination Coverage Survey design tables. These assumptions are consistent with empirical findings from similar large‐scale EPI surveys in the Eastern Mediterranean Region and provide conservative estimates to ensure precision across provinces. The ICC of 0.33 reflects the expected degree of homogeneity within clusters; higher values increase the design effect and therefore widen confidence intervals for a given sample size. This effect is particularly pronounced in rural areas, where clusters (health‐house catchments) tend to exhibit greater similarity in socioeconomic conditions, cultural norms, health‐seeking behaviours, and access to services, leading to potentially higher intra‐cluster correlation and reduced precision compared with urban clusters. To mitigate this, the study deliberately oversamples smaller and more rural strata, applies a conservative ICC assumption in sample‐size calculations, and will report stratum‐specific design effects and effective sample sizes in all outputs. A brief power analysis indicated that the planned sample (*n* = 9200 children) provides > 90% power to detect a 5‐percentage‐point difference in coverage between Iranian and non‐Iranian subgroups (95% vs. 90%, *α* = 0.05, DEFF = 3.3), confirming the adequacy of the proposed design for subgroup comparisons even after accounting for rural ICC inflation.

**Figure 1:**
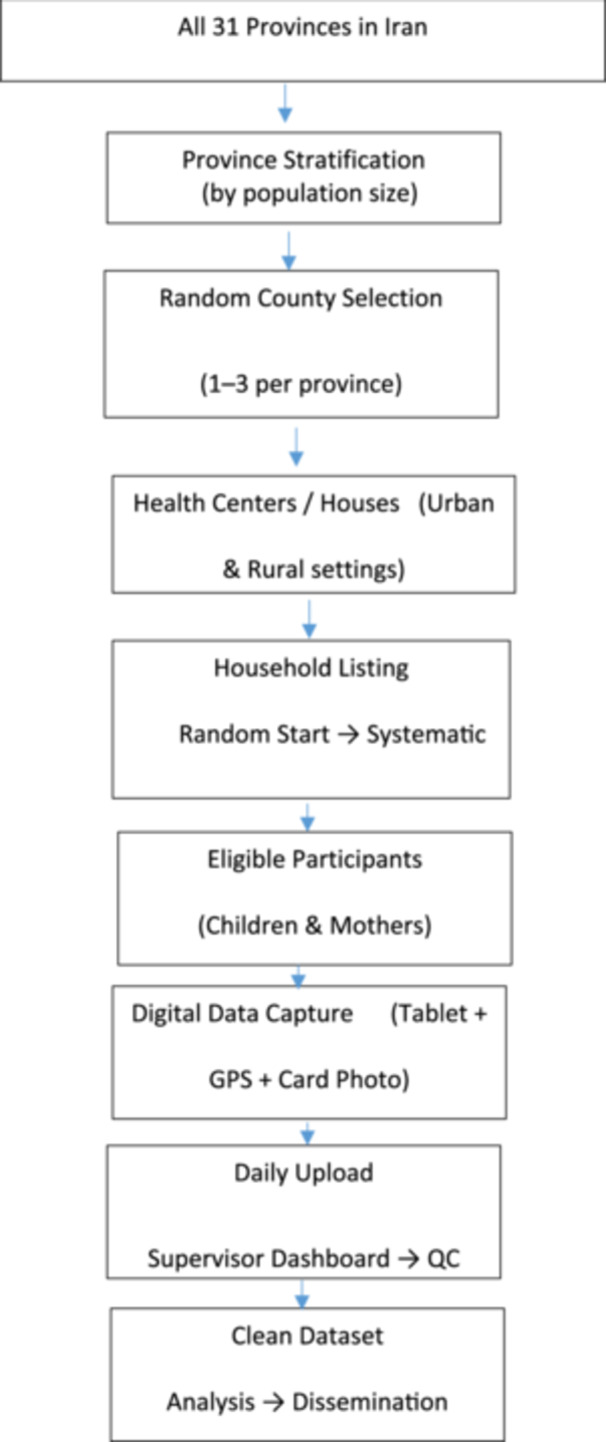
Flowchart of sampling and data flow in the national vaccination coverage survey.

The child survey target sample will be approximately 9200 completed interviews distributed across 1325 clusters with roughly 117 households per cluster, based on stratum‐specific effective sample sizes, design effect assumptions, household eligibility, and nonresponse adjustments. The maternal survey used a single‐stratum design with an effective sample size of 354, inflated for design effect and nonresponse to yield about 2109 completed interviews distributed across 211 clusters, with household listing parameters chosen to locate eligible mothers efficiently. Sample size inputs, including expected coverage, precision, assumed intracluster correlation coefficients, coefficient of variation, design effects, household size, and nonresponse. Within each selected health‐service area or census block, clusters will be selected with probability proportional to size where feasible, and within clusters, the first household will be chosen at random from an updated household listing, followed by systematic sampling to identify subsequent households until cluster targets are met. Where health‐house or health‐centre household lists were unavailable, recent national census block definitions will be used as the sampling frame and all selection steps will be documented to minimize selection bias (Figure 1).

### 2.4 Definition of Clusters and Household Selection Procedure

In this survey, a cluster was defined as the catchment area of a selected health house in rural settings or a health center in urban settings, corresponding approximately to one census block as defined by the national statistical organization. Each cluster, therefore, represents a geographically contiguous area that typically includes 100–150 households.

Within each cluster, households were selected using a systematic random sampling approach. After an updated household listing, the sampling interval (*k*) was calculated as the total number of listed households divided by the number required to meet the cluster target (≈7 eligible households for children under 3 years, or ≈10 for recently delivered mothers). The first household will be chosen randomly between 1 and *k*, and subsequent households will be selected by adding *k* sequentially.

If a selected cluster is inaccessible for safety or logistical reasons, a pre‐defined replacement cluster matched on urban/rural status and population size will be used. All substitutions will be logged, and sensitivity analyses will quantify bias from replacements. Where cluster nonresponse or in‐field attrition threatens subgroup power, contingency sampling will prioritize additional clusters with higher expected non‐Iranian populations. Sensitivity analyses will additionally evaluate the robustness of subpopulation estimates (especially non‐Iranian residents) under alternative weighting schemes. If a selected household is locked or the eligible respondent is absent, two revisits will be attempted at different times of day. If still unsuccessful, the next immediate household in the systematic order will be substituted, and all substitutions will be documented in the cluster log sheet.

Provinces will be represented by the provincial center, and additional randomly selected counties will be chosen according to provincial area. For provinces with an area > 50,000 km^2^, in addition to the provincial center, two additional counties will be randomly selected. For smaller provinces, one additional county (besides the provincial center) will be randomly selected. For each selected county, after coordination with the corresponding Medical University, two urban health‐centers and two rural health houses will be randomly selected as primary sampling units for household listing and cluster selection. Where a selected county falls under the jurisdiction of another Medical University, formal coordination and data‐collection agreements will be established with that university prior to fieldwork.

### 2.5 Data Collection

Quantitative questionnaires will be adapted from the WHO Vaccination Coverage Survey reference instruments and the national 2019 cluster survey and will be translated and culturally adapted into Farsi with content validity assessment [10]. The BeSD core questionnaires and modules will be incorporated to capture behavioral and social determinants of vaccination, including confidence, social processes, practical access, and motivation domains [14]. Data collection will be digital using open‐source mobile data collection platforms with full offline capability, photo capture of home‐based records linked to household and respondent identifiers, global positioning system (GPS) coordinates for geo‐validation, and embedded validation checks and skip logic to reduce entry errors.

Vaccination status will be determined using a hierarchical decision algorithm (1): documented evidence from photographed home‐based records, (2) validated electronic immunization registry records matched by national identifier or household identifiers when linkage is permitted, and (3) caregiver recall when no documentation exists. When card dates are missing or ambiguous, we will attempt registry lookup. If the registry is unavailable, dates will be treated as partially missing and handled in analysis using multiple imputation. A validation sub‐study in about 10% of clusters will compare survey data with facility/electronic records to estimate sensitivity, specificity, and kappa agreement. We will report kappa statistics and apply correction factors where misclassification is systematic [10].

### 2.6 Qualitative Component

The qualitative strand will complement the quantitative survey to explore behavioral, social, and contextual determinants of vaccination. Relying solely on structured survey data would limit the study to statistical associations and prevalence estimates without revealing the underlying mechanisms, cultural nuances, or systemic barriers that drive incomplete or delayed vaccination, information that is indispensable for translating coverage gaps into actionable, equity‐focused interventions. A thematic analysis will be conducted based on the four BeSD domains (i.e., confidence, social processes, practical access, and motivation), which are consistent with the COM‐B framework and the Health Belief Model.

About 30–40 participants, including parents, healthcare workers, community leaders, and policymakers, will be recruited through purposeful sampling. Additional attention will be given to specific subgroups, such as mothers without vaccination cards, and health staff working in rural and per‐urban areas, to ensure the inclusion of diverse perspectives and experiences. Thematic saturation will not be reached until recruitment continues.

MAXQDA 2024 software will be used to audio‐record interviews, transcribe them verbatim, and analyze them for data collection and analysis. The data will be coded by two independent researchers, and intercoder reliability will be evaluated to ensure consistency in coding. To validate the accuracy of interpretations, the qualitative research team will conduct peer debriefing and use member checking with selected participants.

A triangulation matrix will be used to link qualitative findings to quantitative results, allowing for the identification of convergent, complementary, or divergent evidence across data sets. The interpretation of quantitative disparities in vaccination coverage (e.g., by region, nationality, or sex) will be informed by qualitative insights and will help to explain behavioral and structural mechanisms that underlie incomplete immunization.

### 2.7 Mixed‐Methods Integration

The study will use a convergent parallel integration model that involves collecting quantitative and qualitative data simultaneously and analyzing independently, followed by systematic triangulation to ensure a true mixed‐methods design (Table 2). This approach is chosen over a survey‐only design because quantitative coverage estimates, while precise and generalizable, are limited in explanatory power; they identify “who” is under‐vaccinated but cannot elucidate the complex, context‐specific reasons “why.” The qualitative findings provide in‐depth and narrative insights required to interpret quantitative disparities and generate practical solutions.

**Table 2:** Framework for quantitative–qualitative integration in the mixed‐methods design.

Stage	Quantitative component	Qualitative component	Integration purpose
Data collection	Household survey estimating coverage, dropout, and timeliness	In‐depth interviews on vaccine confidence, access, and motivation	Contextualize patterns observed in coverage data
Data analysis	Weighted analysis by province, nationality, and sex	Thematic coding by BeSD domains	Identify behavioral or system‐level causes of quantitative disparities
Interpretation	Identify low‐coverage groups	Extract dominant themes per group	Merge findings using a joint display table linking numeric gaps to explanatory narratives
Policy translation	Statistical dashboards for EPI	Policy briefs synthesizing behavioral insights	Develop integrated equity‐focused recommendations

**Figure 2:**
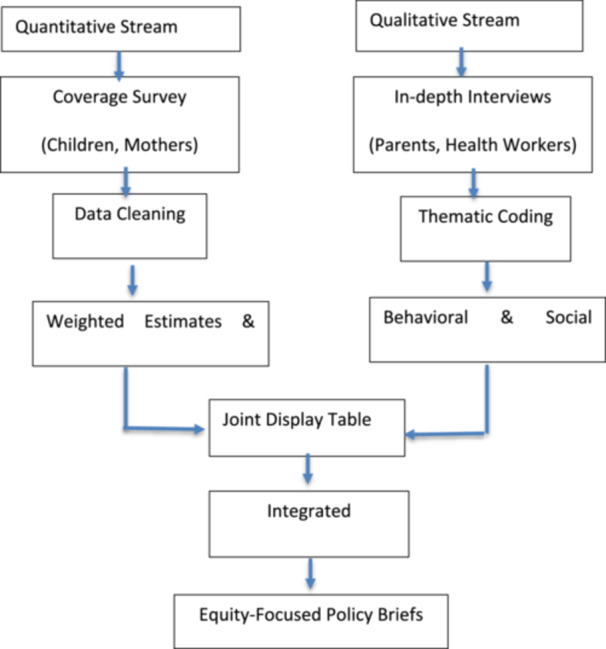
Mixed‐methods integration pathway.

This integration approach allows the study to move beyond descriptive statistics, linking “who is unvaccinated” (quantitative) with “why” (qualitative), thus improving programmatic decision‐making. The corresponding data integration pathway is summarized in Figure 2.

### 2.8 Quality Assurance and Monitoring

Data quality assurance will include pre‐field training and standardization of interviewers and supervisors, pilot testing of instruments, on‐site supervisory review, random back‐checks, automated server‐side validation rules, and daily supervisory dashboards for remote monitoring and rapid feedback loops. If a cluster becomes inaccessible for safety or operational reasons, the steering group will document the reason and either postpone data collection until access is restored or select a pre‐defined replacement cluster matched by urban/rural status and population size; all substitutions will be recorded and reported, and sensitivity analyses will explore the impact of any replacements [8]. Additionally, a validation sub‐study will be implemented in approximately 10% of clusters, where vaccination status from survey responses (card and recall) will be cross‐checked against electronic health registry records. Discrepancies will be analyzed to estimate misclassification and recall error rates. This sub‐component will enable the calculation of sensitivity, specificity, and positive predictive values of survey‐reported vaccination data, enhancing overall validity and providing correction factors for future surveys.

### 2.9 Operational Teams, Pilot, and Training

Fieldwork will be conducted by county‐based teams under the supervision of university and county coordinators. In small counties, data collection will be conducted by one two‐person team; in larger counties, 2–3 teams will be deployed depending on workload and distance. A pilot will be conducted in selected counties to test sampling procedures, digital tools, cluster allocation rules, and the logistic assumptions. Pilot outcomes will be used to finalize cluster sizes and the number of teams per county. Training will combine a 1‐day virtual pre‐deployment session for coordinators with an in‐person practical workshop for field teams covering household listing, consent procedures, photographing cards, and data entry quality checks.

### 2.10 Statistical Analysis

Primary indicators are defined: full vaccination for children 24–35 months is receipt of BCG, three doses of pentavalent (DTP–HepB–Hib), three doses of OPV/IPV as per schedule, and MMR1/MMR2 as age‐appropriate. Timeliness is defined as doses received within the WHO‐recommended and national EPI age windows. Dropout is the percent difference between DTP1 and DTP3. Zero‐dose is no documented receipt of any vaccine.

Analyses will account for the complex survey design by applying sampling weights, clustering, and stratification in all estimations and will compute crude and valid coverage, dropout rates, timeliness, and missed opportunities for simultaneous vaccination using methods recommended in the WHO manual [10]. Variance estimation will incorporate the observed ICC and design effects, with particular attention to rural strata where higher clustering is anticipated. Stratum‐specific ICC values will be reported to allow readers to assess the impact on precision. Weighting and variance estimation procedures will be documented and implemented in Stata (svyset + svy: commands), and reproducible scripts will be provided; for R users the survey package (svydesign + svymean/svyglm) is specified as an alternative. Variance estimation will use the Taylor linearization method by default. Multivariable regression models will be design‐adjusted logistic or multinomial models. The analysis plan specifies candidate predictors and a priori interactions to be tested (notably nationality × urban/rural). Model building will use purposeful selection and check for collinearity. Interaction terms will be reported when statistically meaningful.

The Stata software (v18) will be used for all quantitative analyses. Weighted estimates, design effects, and confidence intervals are calculated using recommended standardized procedures for Stata. Estimating vaccine‐specific coverage rates, dropouts, and timeliness will be the objective of primary analyses, which will be disaggregated by province, sex, residence, and nationality. Multivariable logistic regression models will be used in secondary analyses to identify predictors of incomplete vaccination (e.g., missed or delayed doses), taking into account demographic, socioeconomic, and BeSD‐related factors. The use of design‐adjusted Wald or F‐tests will be utilized to test subgroup differences (such as Iranian vs. non‐Iranian). Qualitative data will be coded and analyzed in MAXQDA 2024 using an inductive–deductive thematic approach aligned with the COM‐B framework. Confidence intervals will be calculated using methods appropriate for complex samples and extreme proportions (e.g., Wilson or other recommended asymmetrical methods), and design effects and intracluster correlation coefficients will be estimated from the survey data to inform future survey planning. Subgroup analyses will produce disaggregated estimates by province, urban/rural residence, sex, and nationality (Iranian vs. non‐Iranian), and sensitivity analyses, multiple imputation, and nonresponse adjustments will be used to assess the impact of missing data and recall‐based information on coverage estimates. In addition, dedicated sensitivity analyses will examine the robustness of subpopulation estimates, particularly for non‐Iranian residents, by comparing primary weighted results with (i) alternative weighting schemes that simulate proportional rather than disproportionate stratification, (ii) complete‐case analyses excluding replacement clusters, and (iii) scenarios that vary the assumed intracluster correlation for non‐Iranian subgroups. These analyses will be reported transparently to confirm that any subtle biases introduced by the multistage disproportionate design do not materially affect the main conclusions. Incomplete vaccination dates will be addressed via multiple imputation under plausible missingness assumptions; imputed data sets will be analyzed with survey‐weighted procedures and pooled estimates reported following Rubin's rules.

For qualitative data, audio‐recorded in‐depth interviews will be transcribed verbatim, translated where necessary, and analyzed thematically using an inductive–deductive approach aligned with the BeSD analytical framework to identify behavioral and social drivers, structural barriers, and context‐specific enablers of vaccination uptake. Triangulation of quantitative and qualitative findings will be performed to contextualize coverage estimates and to generate actionable recommendations for targeted interventions and policy adjustments. The qualitative component will include a codebook matrix and a statement on saturation criteria. We will produce joint tables that integrate quantitative disparities with qualitative explanatory themes. Intercoder reliability will be quantified [14, 15].

Using GPS data collected for each cluster, spatial mapping and hotspot analyses will be performed to visualize geographic disparities in coverage and dropout. These spatial results will support targeted interventions and equity‐based program planning. Final reporting will follow the WHO Vaccination Coverage Survey report checklist and Preferred Reporting Items for Complex Sample Survey Analysis (PRICSSA) recommendations for transparent presentation of complex survey analyses, and results will be shared with national and provincial health authorities, partners, and communities through policy briefs, a detailed technical report in Farsi and English, and peer‐reviewed manuscripts (Supporting Information S2: Appendix 2) [10, 15].

### 2.11 Ethical and Operational Considerations

The survey will receive ethics approval from relevant institutional review boards and will obtain informed verbal consent, documented electronically with a time‐stamp, interviewer ID, and GPS coordinates, or written consent where required, in accordance with WHO ethical guidance for public health surveys and national regulations [10]. Prior to fieldwork in each county, the county coordinator will conduct a brief stakeholder notification to local health authorities and community leaders explaining the survey purpose and data protections, and documentation of this notification will be archived. Participation will be voluntary, and respondents may decline or withdraw without consequence. Data protection measures will be applied to ensure confidentiality and compliance with Iran's data protection provisions. Standard WHO survey logistics will be followed in the field implementation. Provincial health authorities will coordinate access to remote or dense areas to ensure safety and inclusion. To avoid access restrictions, the identification of substitute clusters will be done beforehand. Local bilingual interviewers will be recruited in provinces with high non‐Iranian populations to improve communication and trust. Digital data will be encrypted in transit and stored on secure servers with access restricted to authorized study personnel, and all personal identifiers will be removed from analytical data sets to protect participant confidentiality in line with international and national regulations. Photographed cards will be hashed, encrypted, and stored separately from identifiable metadata. Access is role‐based, and image filenames will use study IDs only. Data transfer will use encrypted channels and backups. A data management plan describing retention and controlled sharing is included.

## 3 Discussion

The present protocol describes a large, mixed‐methods, nationally representative vaccination coverage and BeSD assessment designed to update and validate Iran's routine immunization estimates and to identify equity gaps and programmatic barriers. National cluster survey data collected in 2019 showed very high overall coverage but revealed persistent gaps among non‐Iranian residents and a small number of provinces with coverage below the 95% threshold, demonstrating the need for periodic, rigorous reassessments of coverage and equity [5]. The WHO guidance emphasizes that high‐quality coverage surveys are the gold standard for validating administrative estimates and for minimizing biases of routine reporting systems, and the present design follows those best practices while adding BeSD tools and digital data collection to strengthen validity and program relevance [10].

Recovery of routine immunization after the COVID‐19 pandemic has been uneven, and the number of zero‐dose and incompletely vaccinated children remains a major global concern [16]. There were increases in vaccine hesitancy and access barriers following the pandemic and during socioeconomic shifts, reinforcing the need to pair quantitative coverage measurement with systematic investigation of behavioral and social determinants of uptake [17]. By integrating BeSD modules into the household survey and by planning qualitative interviews, this protocol will produce actionable, context‐specific information about why children miss doses, distinguishing demand‐side issues (e.g., hesitancy, knowledge, acceptability) from supply‐side constraints (e.g., stockouts, access) [14].

A strength of this protocol is its attention to data quality and to triangulating multiple information sources. Administrative reports are convenient but can both over‐ and under‐estimate coverage because of reporting pressures. A recent mixed‐method investigation has shown systematic mechanisms that produce over‐reporting and misclassification in routine immunization systems [18]. By prioritizing documented evidence (cards and electronic health records), photographing home‐based records when available, and building automatic validation and supervisory re‐checks into digital data capture, the study aims to reduce information bias and to quantify the magnitude and direction of discrepancies between administrative and survey estimates. The inclusion of cluster‐level design effects, planned calculation of actual design effects from survey data, and stratified sampling to over‐represent smaller, potentially marginalized provinces will strengthen the precision and interpretability of subnational estimates.

Our focus on migrants, refugees, and non‐Iranian residents addresses a widely documented vulnerability. Studies showed that migrant populations frequently experience multiple barriers (e.g., legal, linguistic, logistical, informational) to routine vaccination and often have lower uptake than host populations [19, 20]. In this regard, the national findings from 2019 survey in Iran suggested lower full immunization among non‐Iranian children, highlighting that focusing on these groups is not only equitable but epidemiologically critical to prevent immunity gaps and local outbreaks [5]. The planned purposive qualitative interviews with caregivers, community leaders, and health staff are likely to reveal the discriminating mix of barriers and facilitators among migrant and to inform culturally and legally appropriate catch‐up strategies.

Another innovation of the protocol is its readiness to document and monitor the rollout of newly introduced vaccines (pneumococcal conjugate and rotavirus vaccines) and to evaluate their uptake relative to the established EPI. Global and regional experience showed that introduction of new antigens requires careful monitoring of coverage, timeliness, and missed‐opportunity patterns to ensure that new vaccines reach the same equity and quality standards as traditional EPI vaccines; surveillance networks and targeted coverage surveys have proven useful in guiding introduction strategies [21, 22]. Our combined card/electronic‐registry approach will provide the evidence needed to guide program adjustments during the initial years post‐introduction.

There are some limitations that should be acknowledged. Household‐based surveys face nonresponse, recall bias when cards are unavailable, and potential cluster inaccessibility. The protocol includes mitigation measures, such as revisits, multiple information sources, sensitivity analyses, and transparent documentation of substitutions or missing clusters, yet some residual uncertainty will remain and will be communicated. In addition, while digital data capture and potential linkage to electronic immunization registries can improve timeliness and traceability, implementation challenges, such as workforce capacity, parallel systems, infrastructure and sustainability, have been observed in multiple low‐ and middle‐income country evaluations [23].

The qualitative findings will inform the design of vaccination outreach programs. For example, community‐level barriers (such as geographic isolation, transportation costs, or mistrust in formal health services) and cultural factors (including traditional beliefs about vaccine timing, family decision‐making roles, or stigma associated with certain vaccines) will be translated into context‐specific, equity‐focused interventions. These may include mobile outreach teams co‐designed with community leaders, culturally adapted communication materials in local languages and dialects, peer‐led education sessions in migrant settlements, or integration of vaccination services with existing community events and religious gatherings. By linking these qualitative insights with quantitative coverage gaps, the study will provide program managers with concrete blueprints for designing and piloting more effective, acceptable, and sustainable outreach strategies.

### 3.1 Innovation of This Protocol

The study integrates three validation streams: photography of home‐based vaccination cards linked to household identifiers, GPS coordinates for each household and cluster, and linkage to national electronic immunization registries where permitted to produce higher‐fidelity coverage estimates than studies relying on single sources. Combining card photographs with GPS enables audit trails and geo‐validation to detect implausible spatial patterns and to re‐visit households when data appear inconsistent; electronic record linkage allows reconciliation of card/recall discrepancies and rapid estimation of registry completeness. The protocol also prioritizes migrants and non‐Iranian residents, stratified provincial comparisons, and monitoring uptake of newly introduced vaccines (pneumococcal conjugate and rotavirus), ensuring that equity and new‐vaccine integration are central outcomes.

## 4 Conclusions

This mixed‐methods national survey will generate policy‐relevant, high‐quality evidence that validates administrative coverage estimates, identifies pockets of under‐immunization, elucidates behavioral and social drivers of missed vaccination, and monitors the early impact of new vaccine introductions. The findings will directly support national policymakers and EPI program managers in designing targeted, equity‐focused interventions—particularly for integrating newly introduced vaccines such as pneumococcal conjugate and rotavirus—by providing disaggregated coverage data, dropout and timeliness indicators, and actionable insights into demand‐ and supply‐side barriers. This evidence will enable data‐driven adjustments to outreach strategies, resource allocation, and communication approaches, thereby strengthening Iran's EPI resilience, closing equity gaps, and ensuring sustained high uptake of both established and new vaccines in the post‐pandemic era. The study has strong potential to inform targeted programmatic responses and to strengthen Iran's EPI resilience in the post‐pandemic era.

## Author Contributions

**Seyed Aria Nejadghaderi:** writing – original draft, writing – review and editing, conceptualization, methodology, software, resources, investigation, and visualization. **Paria Dehesh:** visualization, writing – original draft, writing – review and editing, conceptualization, methodology, software, investigation, resources, and project administration. **Mehran Nakhaeizadeh:** writing – original draft, writing – review and editing, conceptualization, methodology, visualization, and software. **Mohammadreza Rajabalipour:** writing – original draft, writing – review and editing, conceptualization, and methodology. **Faeze Tahrudi:** writing – original draft, writing – review and editing, methodology, and investigation. **Naser Nasiri:** writing – original draft, writing – review and editing. **Seyed Mohsen Zahraei:** writing – original draft, writing – review and editing, methodology, supervision, project administration, and validation. **Hamid Sharifi:** writing – original draft, writing – review and editing, conceptualization, project administration, supervision, validation, and methodology. **AliAkbar Haghdoost:** methodology, writing – review and editing, writing – original draft, conceptualization, project administration, supervision, resources, validation, and visualization.

## Ethics Statement

The Ethics Committee of Kerman University of Medical Sciences, Kerman, Iran, approved the present study (ethics code: IR.KMU.REC.1404.211).

## Consent

The authors have nothing to report.

## Conflicts of Interest

The authors declare no conflicts of interest.

## Transparency Statement

The corresponding authors Paria Dehesh and AliAkbar Haghdoost affirm that this manuscript is an honest, accurate, and transparent account of the study being reported; that no important aspects of the study have been omitted; and that any discrepancies from the study as planned (and, if relevant, registered) have been explained.

## Supporting information

Supporting File 1

Supporting File 2

## Data Availability

Data sharing is not applicable to this article as no data sets were generated or analyzed during the current study.
