# Supplementary material for: A Protocol of National Mixed‐Methods Assessment of Childhood and Maternal Immunization
Source: Health Sci Rep. 2026 Jun 7;9(6):e72566. doi: 10.1002/hsr2.72566 (PMC13242692; doi:10.1002/hsr2.72566)
Supplement: Supplementary file 1 — Supporting File 1 [file HSR2-9-e72566-s002.docx]

**Appendix 1. Sample size calculation.**

The details of sample size calculation for children are as follow:

13 province exist in stratum 1 we use below information to calculate sample size

| **Step** | **Letter** | **Quantity** | **Inputs** | **(Specify Inputs)** | **Output using Table or Equation** |
| --- | --- | --- | --- | --- | --- |
| 1 | (A) | Number of Strata (NStrata) | (no inputs) | |  |
| 2 | (B) | Effective Sample Size (ESS) – Estimation with Desired Precision | Expected coverage | 0.95 | 70 |
|  |  |  | Precision level | ±10% |  |
|  |  | Effective Sample Size (ESS) – Classification | Programmatic threshold | - |  |
|  |  |  | Delta & Direction | - |  |
|  |  |  | Alpha | - |  |
|  |  |  | Power | - |  |
| 3 | (C) | Design Effect (DEFF) | *m* | 7 | 3.27 |
|  |  |  | ICC | 0.33 |  |
|  |  |  | CVw | 0.3 |  |
| 4 | (D) | Number of Households to Visit to Find an Eligible Child (NHH to find eligible child) | (no inputs) | | 15 |
| 5 | (E) | Nonresponse (INonresponse) | PHH eligible and not respond | 5% | 1.11 |

**The summary table for stratum 1:**

| **Description** | **Value** |
| --- | --- |
| Number of completed surveys | 2,982.9 |
| Households to visit | 49,721.4 |
| Clusters per stratum | 33 |
| Total clusters | 429 |
| Households per cluster | 116.55 |

13 province exist in stratum 2 we use below information to calculate sample size

| **Step** | **Letter** | **Quantity** | **Inputs** | **(Specify Inputs)** | **Output using Table or Equation** |
| --- | --- | --- | --- | --- | --- |
| 1 | (A) | Number of Strata (NStrata) | (no inputs) | |  |
| 2 | (B) | Effective Sample Size (ESS) – Estimation with Desired Precision | Expected coverage | 0.95 | 93 |
|  |  |  | Precision level | ±8% |  |
|  |  | Effective Sample Size (ESS) – Classification | Programmatic threshold | - |  |
|  |  |  | Delta & Direction | - |  |
|  |  |  | Alpha | - |  |
|  |  |  | Power | - |  |
| 3 | (C) | Design Effect (DEFF) | *m* | 7 | 3.27 |
|  |  |  | ICC | 0.33 |  |
|  |  |  | CVw | 0.3 |  |
| 4 | (D) | Number of Households to Visit to Find an Eligible Child (NHH to find eligible child) | (no inputs) | | 15 |
| 5 | (E) | Nonresponse (INonresponse) | PHH eligible and not respond | 10% | 1.11 |

**The summary table for stratum 2:**

| **Description** | **Value** |
| --- | --- |
| Number of completed surveys | 3,957.57 |
| Households to visit | 65,963.4 |
| Clusters per stratum | 44 |
| Total clusters | 572 |
| Households per cluster | 116.55 |

Four province exist in stratum 3 we use below information to calculate sample size

| **Step** | **Letter** | **Quantity** | **Inputs** | **(Specify Inputs)** | **Output using Table or Equation** |
| --- | --- | --- | --- | --- | --- |
| 1 | (A) | Number of Strata (NStrata) | (no inputs) | |  |
| 2 | (B) | Effective Sample Size (ESS) – Estimation with Desired Precision | Expected coverage | 0.95 | 132 |
|  |  |  | Precision level | ±6% |  |
|  |  | Effective Sample Size (ESS) – Classification | Programmatic threshold | - |  |
|  |  |  | Delta & Direction | - |  |
|  |  |  | Alpha | - |  |
|  |  |  | Power | - |  |
| 3 | (C) | Design Effect (DEFF) | *m* | 7 | 3.27 |
|  |  |  | ICC | 0.33 |  |
|  |  |  | CVw | 0.3 |  |
| 4 | (D) | Number of Households to Visit to Find an Eligible Child (NHH to find eligible child) | (no inputs) | | 15 |
| 5 | (E) | Nonresponse (INonresponse) | PHH eligible and not respond | 10% | 1.11 |

**The summary table for stratum 3:**

| **Description** | **Value** |
| --- | --- |
| Number of completed surveys | 1,726.56 |
| Households to visit | 28,741.8 |
| Clusters per stratum | 62 |
| Total clusters | 248 |
| Households per cluster | 116.55 |

One province exist in stratum 4 we use below information to calculate sample size

| **Step** | **Letter** | **Quantity** | **Inputs** | **(Specify Inputs)** | **Output using Table or Equation** |
| --- | --- | --- | --- | --- | --- |
| 1 | (A) | Number of Strata (NStrata) | (no inputs) | |  |
| 2 | (B) | Effective Sample Size (ESS) – Estimation with Desired Precision | Expected coverage | 0.95 | 162 |
|  |  |  | Precision level | ±5% |  |
|  |  | Effective Sample Size (ESS) – Classification | Programmatic threshold | - |  |
|  |  |  | Delta & Direction | - |  |
|  |  |  | Alpha | - |  |
|  |  |  | Power | - |  |
| 3 | (C) | Design Effect (DEFF) | *m* | 7 | 3.27 |
|  |  |  | ICC | 0.33 |  |
|  |  |  | CVw | 0.3 |  |
| 4 | (D) | Number of Households to Visit to Find an Eligible Child (NHH to find eligible child) | (no inputs) | | 15 |
| 5 | (E) | Nonresponse (INonresponse) | PHH eligible and not respond | 10% | 1.11 |

**The summary table for stratum 4:**

| **Description** | **Value** |
| --- | --- |
| Number of completed surveys | 529.74 |
| Households to visit | 8818.14 |
| Clusters per stratum | 76 |
| Total clusters | 76 |
| Households per cluster | 116.55 |

**The data and parameters for each stratum:**

| **Stratum** | **A** | **B (HH size)** | **C (Target interviews)** | **D (DEFF)** | **E (adj.)** | **m** |
| --- | --- | --- | --- | --- | --- | --- |
| 1 | 13 | 3.27 | 70 | 15 | 1.11 | 7 |
| 2 | 13 | 3.27 | 93 | 15 | 1.11 | 7 |
| 3 | 4 | 3.27 | 132 | 15 | 1.11 | 7 |
| 4 | 1 | 3.27 | 162 | 15 | 1.11 | 7 |

**The final table for sample size calculation of children:**

| **Stratum** | **Ncs** | **NHH to Visit** | **Clusters/Stratum** | **Total Clusters** | **HHs/Cluster** |
| --- | --- | --- | --- | --- | --- |
| 1 | 2982.9 | 49,721.4 | 32.7 ≈ **33** | **429** | 116.55 |
| 2 | 3960.5 | 65,963.4 | 43.5 ≈ **44** | **572** | 116.55 |
| 3 | 1727.3 | 28,741.8 | 61.6 ≈ **62** | **248** | 116.55 |
| 4 | 529.7 | 8818.14 | 75.7 ≈ **76** | **76** | 116.55 |
| Total | 9200.4 | 153244.7 | 215 | 1325 | 466.2 |

For the maternal survey, a single‐stratum design yields an effective sample size of 354 interviews, which translates into about 2,109 completed surveys once adjustments for design effect, household eligibility, and nonresponse are applied. Achieving this requires visiting approximately 70,208 households distributed across 211 clusters, each encompassing an average of 333 households.

The details of sample size calculation for maternal vaccination are as follow:

| **Step** | **Letter** | **Quantity** | **Inputs** | **(Specify Inputs)** | **Output using Table or Equation** |
| --- | --- | --- | --- | --- | --- |
| 1 | (A) | Number of Strata (NStrata) | (no inputs) | |  |
| 2 | (B) | Effective Sample Size (ESS) – Estimation with Desired Precision | Expected coverage | 0.95 | 354 |
|  |  |  | Precision level | ±3% |  |
|  |  | Effective Sample Size (ESS) – Classification | Programmatic threshold | - |  |
|  |  |  | Delta & Direction | - |  |
|  |  |  | Alpha | - |  |
|  |  |  | Power | - |  |
| 3 | (C) | Design Effect (DEFF) | *m* | 10 | 5.96 |
|  |  |  | ICC | 0.33 |  |
|  |  |  | CVw | 0.7 |  |
| 4 | (D) | Number of Households to Visit to Find an Eligible Child (NHH to find eligible child) | (no inputs) | | 30 |
| 5 | (E) | Nonresponse (INonresponse) | PHH eligible and not respond | 10% | 1.11 |

**The data and parameters are provided below:**

| **Parameter** | **Value** |
| --- | --- |
| Number of strata (A) | 1 |
| Average household size (B) | 5.96 |
| Target interviews (C) | 354 |
| Design effect (D) | 30 |
| Adjustment factor (E) | 1.11 |
| Interviews per cluster (m) | 10 |

**The final table for sample size calculation of maternal vaccination:**

| **Description** | **Value** |
| --- | --- |
| Number of completed surveys | 2,108.64 |
| Households to visit | 70,207.92 |
| Total clusters | 211 |
| Households per cluster | 333 |
