# Supplementary material for: A Protocol of National Mixed‐Methods Assessment of Childhood and Maternal Immunization
Source: Health Sci Rep. 2026 Jun 7;9(6):e72566. doi: 10.1002/hsr2.72566 (PMC13242692; doi:10.1002/hsr2.72566)
Supplement: Supplementary file 2 — Supporting File 2 [file HSR2-9-e72566-s001.docx]

**Appendix 2.** Preferred Reporting Items for Complex Sample Survey Analysis (PRICSSA) checklist.

| PRICSSA item | Description | Page |
| --- | --- | --- |
| 1.1 Data collection dates | Describe the survey’s data collection dates (e.g., range) to provide historical context that could affect survey responses and nonresponse. |  |
| 1.2 Data collection mode(s) | Describe the survey’s data collection mode(s). Data collection mode can affect survey responses (e.g., to sensitive questions), including nonresponse, and a survey’s data collection mode may change over time (e.g., during the COVID-19 pandemic). |  |
| 1.3 Target population | State the target population the survey was designed to represent and describe all weighted estimates with respect to this target population. |  |
| 1.4 Sample design | Describe the survey’s sample design, including information about stratification, cluster sampling, and unequal probabilities of selection. |  |
| 1.5 Survey response rate(s) | State the survey’s response rate and how it was calculated. |  |
| 2.1 Missingness rates | Report rates of missingness for variables of interest and models, and describe any methods (if any) for dealing with missing data (e.g., multiple imputation). |  |
| 2.2 Observation deletion | State whether any observations were deleted from the dataset. If observations were deleted, provide a justification. Note: It is best practice to avoid deleting cases and use available subpopulation analysis commands no matter what variance estimation method is used. |  |
| 2.3 Sample sizes | Include unweighted sample sizes for all weighted estimates. |  |
| 2.4 Confidence intervals/standard errors | Include confidence intervals or standard errors when reporting all estimates to inform the reliability/precision of each estimate. |  |
| 2.5 Weighting | State which analyses were weighted and specify which weight variables were used in analysis. |  |
| 2.6 Variance estimation | Describe the variance estimation method used in the analysis and specify which design variables (e.g., PSU/stratum, replicate weights) were used. |  |
| 2.7 Subpopulation analysis | Describe the procedures used for conducting subpopulation analyses (e.g., Stata’s “subpop” command, SAS’s “domain” command). |  |
| 2.8 Suppression rules | State whether or not a suppression rule was followed (e.g., minimum sample size or relative standard error). |  |
| 2.9 Software and code | Report which statistical software was used, comprehensively describe data management and analysis in the manuscript, and provide all statistical software code. |  |
| 2.10 Singleton problem (as needed) | Taylor Series Linearization requires at least two PSUs per stratum for variance estimation. Sometimes an analysis is being performed and there is only a single PSU in a stratum. There are several possible fixes to this problem, which should be detailed if the singleton problem is encountered. |  |
| 2.11 Public/restricted data (as needed) | If applicable, state whether the public use or restricted version of the dataset was analyzed. |  |
| 2.12 Embedded experiments (as needed) | If applicable, provide information about split sample embedded experiments (e.g., mode of data collection or varying participant incentives) and detail whether experimental factors were accounted for in the analyses. |  |
